# Disparities in Neighborhood Characteristics among U.S. Children with Secondhand and Thirdhand Tobacco Smoke Exposure

**DOI:** 10.3390/ijerph19074266

**Published:** 2022-04-02

**Authors:** E. Melinda Mahabee-Gittens, Rebecca A. Vidourek, Keith A. King, Ashley L. Merianos

**Affiliations:** 1 Division of Emergency Medicine, Cincinnati Children’s Hospital Medical Center, University of Cincinnati College of Medicine, Cincinnati, OH 45229, USA; 2 School of Human Services, University of Cincinnati, Cincinnati, OH 45221, USA; rebecca.vidourek@uc.edu (R.A.V.); keith.king@uc.edu (K.A.K.); ashley.merianos@uc.edu (A.L.M.)

**Keywords:** children, neighborhoods, tobacco smoke exposure, secondhand smoke, thirdhand smoke

## Abstract

(1) Background: Home tobacco smoke exposure (TSE) and negative neighborhood characteristics adversely affect children’s overall health. The objective was to examine the associations of child TSE status and neighborhood characteristics among U.S. school-aged children. (2) Methods: We conducted a secondary analysis of the 2018–2019 National Survey of Children’s Health (NSCH) data including 17,300 U.S. children ages 6–11 years old. We categorized children’s home TSE status into: (a) no TSE: child did not live with a smoker; (b) thirdhand smoke (THS) exposure alone: child lived with a smoker who did not smoke inside the home; and (c) secondhand smoke (SHS) and THS exposure: child lived with a smoker who smoked inside the home. We conducted a series of weighted linear and logistic regression analyses to assess the associations between child TSE status and neighborhood characteristics, adjusting for covariates. (3) Results: Overall, 13.2% and 1.7% of children were exposed to home THS alone and home SHS and THS, respectively. Compared to children with no TSE, children with home THS exposure alone and children with home SHS and THS exposure had a significantly lower total number of neighborhood amenities and children with SHS and THS exposure had a significantly higher total number of detracting neighborhood elements. (4) Conclusions: Children with TSE demonstrate disparities in the characteristics of the neighborhood in which they live compared to children with no TSE. TSE reduction interventions targeted to children with TSE who live in these neighborhoods are warranted.

## 1. Introduction

Children are involuntarily exposed to tobacco smoke when they inhale secondhand smoke (SHS) from active smokers or when they inhale, ingest, or dermally absorb tobacco smoke residue, also called thirdhand smoke (THS), from surfaces or dust in environments in which tobacco products have been previously used [1]. Children’s overall tobacco smoke exposure (TSE) consists of exposure to both SHS and THS if they live with smokers who smoke indoors or exposure to THS alone if they live with smokers who do not smoke indoors. While the prevalence of TSE in children is approximately 38% [2], children who are racially/ethnically diverse, impoverished, live in rented homes, and around more smokers have an even higher prevalence of TSE of up to 54% [3]. Pediatric TSE results in a wide range of infectious and respiratory illnesses and increases children’s risk of diseases during adulthood such as cancer, cardiovascular disease, and obesity [4,5].

Children who live with tobacco smokers are more likely to initiate smoking themselves due to their observations of tobacco use behaviors by others in their homes [6,7]. The neighborhoods in which children live may also contribute to their future risk of experiencing TSE or initiating tobacco use. Specifically, the prevalence of TSE and primary tobacco use are higher in neighborhoods that are racially/ethnically diverse or of lower socioeconomic status, in part due to the higher density of tobacco retailers and stronger tobacco marketing efforts in these neighborhoods [8,9]. Additionally, there is less stringent enforcement of rules prohibiting sales of tobacco products to minors and anti-smoking policies in multiunit housing and public areas in lower-income neighborhoods [10,11], which also contribute to higher TSE and smoking prevalence rates [12,13]. Finally, lower tobacco product prices may drive active smoking prevalence rates in lower-income neighborhoods and in neighborhoods with more youth [14,15], leading to higher passive smoking levels among children.

When considering how children’s neighborhoods may affect their overall health and potential risk for initiating tobacco use in the future, both the physical and social characteristics of the neighborhood should be assessed as this adds context that may explain their future behaviors [16,17,18]. The physical or built environment consists of the man-made, physical surroundings that may influence children’s physical activity, diet, risky behaviors, and future health outcomes [17,18,19]. These characteristics include the availability of physical spaces in which positive activities can occur. Additionally, the neighborhood’s social environment may contribute to children’s health outcomes and includes the presence of detracting elements such as physical disorder that may lead to stress, adverse mental health outcomes, and lack of neighborhood support such as low social cohesion or collective efficacy [17,18,19]. These risk factors have been associated with behavioral issues such as aggressive and delinquent behaviors among children [20,21].

There is limited research on the associations of neighborhood characteristics of children with TSE in comparison to children with no TSE. Such knowledge would provide critical information on the types of neighborhoods in which children with TSE live so that TSE reduction interventions can be provided to families in these areas. Using the social-ecological model of health as the foundation, we sought to examine the individual (i.e., child sociodemographics and TSE status), relationship and community (i.e., neighborhood amenities such as sidewalks or walking paths or recreation centers), and societal (i.e., detracting elements such as poorly kept or rundown housing) level factors of children with TSE and of children without TSE [22]. Thus, our study objectives were to examine the associations of child TSE status with neighborhood amenities and neighborhood detracting elements among U.S. children 6–11 years of age. We hypothesized that children with TSE would have lower neighborhood amenities but higher neighborhood detracting elements compared to children with no TSE.

## 2. Methods

### 2.1. Participants and Procedures

We conducted a secondary analysis of the 2018–2019 National Survey of Children’s Health (NSCH) data that annually measures U.S. children’s physical and emotional health and well-being. NSCH is a cross-sectional survey of 0–17-year-old noninstitutionalized children living in household units located in all 50 U.S. states and the District of Columbia [23,24]. NSCH is conducted by the U.S. Census Bureau in conjunction with funding and administrative direction by the U.S. Health Resources and Services Administration’s Maternal and Child Health Bureau [23,24]. Households were randomly selected and mailed an invitation and a screener questionnaire to be completed by an adult caregiver/parent which identified all children residing in the household; detailed study procedures are described elsewhere [23,24,25,26]. A total of 18,396 6–11-year-olds participated in the respective age-specific topical questionnaire for the 2018–2019 NSCH. For the current study’s analysis, we excluded participants missing data on child TSE status (*n* = 309; 1.7%) and neighborhood variables (*n* = 787; 4.4%). Therefore, our total analytic sample was 17,300 U.S. children ages 6–11 years old. The protocol for the present study was evaluated by a university-based Institutional Review Board (IRB) which determined that since NSCH data is publicly available, this study was not considered to be human subjects research and was thus exempted the study from review.

### 2.2. Measures

#### 2.2.1. Child Home TSE Status

To assess the independent variable of interest, child home TSE status, we analyzed responses to parental assessments, which asked a yes/no question regarding whether their child lived with any household members that smoked tobacco (e.g., combustible cigarettes). The independent variable of interest for this study was children’s home TSE status, which was categorized into the three groups: (1) no TSE: child did not live with a smoker; (2) THS exposure alone: child lived with a smoker who did not smoke inside the home; and (3) SHS and THS exposure: child lived with a smoker who smoked inside the home.

#### 2.2.2. Neighborhood Characteristics

To assess the dependent variables of children’s neighborhood amenities, parents were asked four yes/no questions about whether there were the following amenities in their neighborhoods: (1) “sidewalks or walking paths”; (2) “a park or playground”; (3) “a recreation center, community center, or boys’ and girls’ club”; (4) and “a library or bookmobile” [25,26]. One of the NSCH indicators measures counts how many of the four amenities were present in children’s neighborhoods (i.e., neighborhood contains 0–4 amenities) [27]. Therefore, we assessed the total number of neighborhood amenities present in the children’s neighborhood as a continuous variable, as well as by the four individual neighborhood amenity types as categorical variables (yes, no).

To assess the dependent variables of presence of detracting elements in children’s neighborhoods, parents were asked three yes/no questions about whether there were the following detracting elements in their neighborhoods: (1) “litter or garbage on the street or sidewalk”; (2) “poorly kept or rundown housing”; and (3) “vandalism such as broken windows or graffiti” [25,26]. Another NSCH indicator was presence of detracting neighborhood elements, which measures how many of the three detracting elements were present in children’s neighborhoods (i.e., neighborhood contains 0–3 detracting elements) [27]. Therefore, we assessed the total number of present detracting neighborhood elements as a continuous dependent variable, as well as the three individual detracting neighborhood elements as categorical variables (yes, no).

#### 2.2.3. Covariates

Based on the extant literature on associations of child TSE status and/or neighborhood environment [3,28], the following sociodemographic characteristics were selected as covariates and included in the models. These variables were: child age, sex, race/ethnicity (i.e., non-Hispanic White, non-Hispanic Black, Hispanic, non-Hispanic Other/Multiracial), parent education level (i.e., high school graduate and equivalent or less, some college, college degree or higher), family household structure (i.e., two parents currently married, two parents not currently married, single parent, other or unknown family type), and family federal poverty level (i.e., 0–199%, 200–299%, 300–399%, and 400% or higher). A calculated variable for federal poverty level based on State Children’s Health Insurance Program (SCHIP) income groups was provided by NSCH versus providing family income level to protect household confidentiality [27].

#### 2.2.4. Statistical Analysis

All analyses were conducted using SPSS Complex Samples version 27.0 [27]. Following the 2018–2019 NSCH methodology guidelines [25,26], we applied sampling weights to account for NSCH survey nonresponses, possible sampling frame issues, and to match survey responses with the U.S. child population in both survey years. We calculated descriptive statistics which included raw sample size counts and weighted percentages for all variables including child TSE status, neighborhood characteristics, and the covariates. Weighted chi-square tests were performed to examine the relationships between the categorical sociodemographic covariates and child home TSE status. A weighted one-way analysis of variance (ANOVA) test was performed to examine the relationship between child age and child home TSE status. All covariates, with the exception of child age and child sex, were significantly associated with child TSE status and included in the regression models.

To answer our main study aims, we conducted two weighted multiple linear regression analyses to assess the associations between child TSE status and the total number of neighborhood amenities and the total number of detracting elements present in children’s neighborhoods, while adjusting for the covariates. We present β and 95% confidence intervals (CIs) for the multiple linear regression models. We also conducted seven weighted multivariable logistic regression analyses to assess the associations between child TSE status and the four categorical neighborhood amenities (e.g., sidewalks or walking paths) and the three categorical detracting elements (e.g., litter or garbage), while adjusting for the covariates. We present adjusted odds ratios (AORs) and 95% CIs for the multivariable logistic regression models. A two-sided *p*-value with *p* < 0.05 indicating significance was used to avoid a Type 1 error.

## 3. Results

The average age of the 17,300 children in the analytic sample was 8.56 (Standard Error, SE = 0.03) years, and 50.9% were male, 50.7% were non-Hispanic White, 12.9% were non-Hispanic Black, 11.2% were a non-Hispanic Other race, including Multiracial, and 25.2% were of Hispanic ethnicity (Table 1). A total of 13.2% of children lived with a household smoker with no TSE (*n* = 2278), and 1.7% lived with a smoker with home TSE (*n* = 298).

### 3.1. Sociodemographic Covariates and Child Home TSE Status

There were statistically significant differences in child race/ethnicity, parent education level, family household structure, and family federal poverty level and child home TSE status (see Table 1). For example, a significant difference was found between child race/ethnicity and home TSE status, with the highest percent of non-Hispanic White (57.4%) children living with a smoker with THS exposure alone and the highest percentages of non-Hispanic White (63.6%) and non-Hispanic Black (21.8%) children living with a smoker with SHS and THS exposure.

### 3.2. Child Home TSE Status and Total Number and Types of Neighborhood Amenities

Overall, children lived in neighborhoods with a mean (SE) of 2.67 (0.02) amenities. By home TSE status, children with no TSE had the highest total mean (SE) number of 2.82 (0.03) neighborhood amenities followed by children with THS alone and children with SHS and THS exposure with a mean of 2.56 (0.06) and 2.42 (0.14) amenities, respectively (See Table 2). Multiple linear regression analyses indicated that children’s home TSE status was significantly negatively associated with the total number of neighborhood amenities. Specifically, children with THS exposure alone (β = −0.27, 95% CI = −0.38, −0.15, *p* < 0.001) and children with SHS and THS exposure (β = −0.41, 95% CI = −0.69, −0.13, *p* = 0.005) had a significantly lower total number of amenities present in their neighborhood compared to children with no TSE, while controlling for the sociodemographic covariates. The types of neighborhood amenities are found in Table 3.

#### 3.2.1. Sidewalks or Walking Paths

By home TSE status, 76.3% of children with no TSE, 65.4% of children exposed to THS alone, and 51.4% of children exposed to SHS and THS lived in a neighborhood with sidewalks or walking paths. Multivariable logistic regression model results indicated that compared to children with no TSE, those exposed to THS alone (AOR = 0.71, 95% CI = 0.60, 0.85, *p* < 0.001) and those exposed to SHS and THS (AOR = 0.49, 95% CI = 0.33, 0.72, *p* < 0.001) were less likely to live in a neighborhood with sidewalks or walking paths, while controlling for the sociodemographic covariates.

#### 3.2.2. Park or Playground

By home TSE status, 77.1% of children with no TSE, 66.3% of children exposed to THS alone, and 63.2% children exposed to SHS and THS lived in a neighborhood with a park or playground. Multivariable logistic regression model results indicated that children with THS exposure alone (AOR = 0.71, 95% CI = 0.57, 0.87, *p* = 0.001) were less likely to live in a neighborhood with a park or playground compared to children with no TSE.

#### 3.2.3. Recreation Centers, Community Centers, or Boys’ and Girls’ Clubs

By home TSE status, 51.0% of children with no TSE, 40.0% of children exposed to THS alone, and 31.9% of children exposed to SHS and THS lived in a neighborhood with a recreation center, community center, or boys’ or girls’ club. Multivariable logistic regression model results indicated that children who had home THS exposure alone (AOR = 0.75, 95% CI = 0.62, 0.92, *p =* 0.006) were less likely to live in a neighborhood with a recreation center, community center, or boys’ and girls’ club compared to children with no TSE. Children who had home SHS and THS exposure (AOR = 0.57, 95% CI = 0.38, 0.85, *p =* 0.006) were less likely to live in a neighborhood with a recreation center, community center, or boys’ and girls’ club compared to children with no TSE.

#### 3.2.4. Library or Bookmobile

By home TSE status, 69.8% of children with no TSE, 60.8% of children exposed to THS alone, and 60.5% of children exposed to SHS and THS lived in a neighborhood with a library or bookmobile. Multivariable logistic regression model results indicated that children who had home THS exposure alone (AOR = 0.78 95% CI = 0.63, 0.95, *p =* 0.014) were less likely to live in a neighborhood with a library or bookmobile compared to children with no TSE.

### 3.3. Child Home TSE Status and Total Number and Types of Detracting Neighborhood Elements

By home TSE status, children with no TSE had the lowest total mean (SE) number of 0.43 (0.02) detracting neighborhood elements, followed by children with THS alone and children with SHS and THS exposure with a mean of 0.51 (0.04) and 0.67 (0.10) elements, respectively (See Table 4). Multiple linear regression analyses indicated that children’s home TSE status was significantly associated with the total number of detracting elements in their neighborhood. Specifically, children with SHS and THS exposure (β = 0.24, 95% CI = 0.05, 0.43, *p* = 0.013) had a significantly higher total number of detracting elements present in their neighborhood compared to children with no TSE, while controlling for the sociodemographic covariates. See Table 5 for the specific types of detracting neighborhood elements.

#### 3.3.1. Litter or Garbage

A total of 20.8% (*n* = 2874) of children lived in a neighborhood with litter or garbage. By home TSE status, 19.8% of children with no TSE, 25.0% of children exposed to THS alone, and 35.4% of children exposed to SHS and THS lived in a neighborhood with litter or garbage (Table 5). Multivariable logistic regression model results indicated that children with SHS and THS exposure (AOR = 1.80, 95% CI = 1.10, 2.95, *p* = 0.020) were more likely to live in a neighborhood with litter or garbage compared to children with no TSE.

#### 3.3.2. Poorly Kept or Rundown Housing

By home TSE status, 11.9% of children with no TSE, 18.5% of children exposed to THS alone, and 23.5% of children exposed to SHS and THS lived in a neighborhood with poorly kept or rundown housing. Children who had home THS exposure alone (AOR = 1.39, 95% CI = 1.06, 1.82, *p* = 0.017) and children with SHS and THS exposure (AOR = 1.65, 95% CI = 1.07, 2.54, *p* = 0.023) were more likely to live in a neighborhood with poorly kept or rundown housing compared to children with no TSE.

#### 3.3.3. Vandalism

By home TSE status, 6.9% of children with no TSE, 8.5% of children exposed to THS alone, and 14.0% of children exposed to SHS and THS lived in a neighborhood with vandalism. There were no statistically significant associations between child home TSE and living in a neighborhood with vandalism.

## 4. Discussion

The current study’s main findings suggest that U.S. children 6–11 years old who live with smokers who do not smoke inside the home (i.e., children with THS exposure alone) and children who live with smokers who smoke inside and outside the home (i.e., children with SHS and THS exposure) face in the types of neighborhoods in which they live. Children with TSE were at reduced likelihood of living in neighborhoods in which there are favorable amenities and at increased likelihood of living in neighborhoods with detracting elements such as litter. In addition to these findings, we report sociodemographic disparities in children with home TSE in that children’s home TSE status differed by child race/ethnicity and socioeconomic indicators of parent education level, family household structure, and family federal poverty level. These findings are congruent to other studies which indicate that TSE is more common in children who are non-Hispanic Black race/ethnicity, with parents who have obtained a lower education status, and who are impoverished [2,3]. Specifically, the current study’s rates of THS exposure alone were highest in children who were non-Hispanic White (57.4%) and Hispanic (22.4%), and the rates of SHS and THS exposure were highest in children who were non-Hispanic White (63.6%) and non-Hispanic Black (21.8%). Our results add to the literature by including a measure of THS exposure which allowed us to identify rates of THS exposure based on sociodemographic characteristics nationwide. Concerning socioeconomic status indicators, children who lived in households where parents had lower education, a family structure other than two parents who were currently married, and lower family federal poverty level had higher rates of THS exposure alone and SHS and THS exposure. Other work has found that socioeconomic disparities in TSE persist even after the implementation of public tobacco control laws, suggesting that income-related TSE differences may be due to issues faced by lower-income populations such as financial insecurity, lack of alternate outdoor places to smoke, and lack of knowledge about TSE risks [29,30,31]. These factors should be addressed in future planning of tobacco control interventions and policies, especially in socioeconomically disadvantaged populations.

Regarding children’s neighborhood amenities, we observed that children with home TSE had a significantly lower number of neighborhood amenities. A great deal of research has examined the benefits of living in neighborhoods that have healthy outdoor greenspaces and indoor spaces that promote constructive activities such as sidewalks, parks or playgrounds, recreation centers, and libraries. For example, proximity to these spaces results in increased physical activity and lower obesity rates [19,32,33]. It is possible that children with TSE who live in neighborhoods that have limited greenspace and neighborhood physical areas such as playgrounds may be at increased risk of initiating smoking due to boredom [34]. Increased greenspace is associated with improved overall health and increased smoking cessation efforts in adults [35,36]; thus, providing healthy spaces for children with TSE to spend time may decrease their risk of initiating smoking in the future. Further, increasing outdoor opportunities for children with TSE may decrease their observations of household members smoking and may decrease the health risks associated with TSE; thus potentially decreasing their risk of future smoking initiation [37,38]. Additionally, children’s homes are the number one source for TSE [39] and therefore more time away from the home while engaged in healthy activities may also decrease the health risks associated with TSE.

Children with TSE had a significantly increased number of detracting elements in their neighborhoods. Specifically, children with SHS and THS exposure were nearly two times more likely to live in a neighborhood with litter or garbage, and children with THS exposure alone and children with both SHS and THS exposure were significantly more likely to live in a neighborhood with poorly kept or rundown housing. These detracting neighborhood elements can lead to decreased social cohesion and lower perceived neighborhood safety [17,18,19].

There are numerous strengths of this study including the use of two years of NSCH secondary data that are nationally representative, and the use of measures of built and social environments that have been used in numerous prior studies [40,41,42,43]. There are also limitations that should be noted. The use of cross-sectional data did not allow us to draw causal associations and or to examine longitudinal trends. Child TSE was assessed by parent report, which may have resulted in underreported TSE patterns; however, our results on the associations of the sociodemographic covariates and TSE parallels that of other national research that biochemically validated children’s TSE levels [3]. Future studies should assess child TSE with biomarkers including cotinine, a nicotine metabolite which measures recent TSE [44], and hand nicotine, a measure of THS in children’s environment that serves as a proxy of children’s THS exposure [45]. Further, since this study only examined children in the U.S., future studies should include children living in countries outside of the U.S. as this would provide a more comprehensive understanding of the associations of child TSE status and neighborhood characteristics.

## 5. Conclusions

In conclusion, children with TSE are at increased risk of living in neighborhoods with lower numbers and types of favorable amenities such as sidewalks or walking paths and higher numbers and types of detracting elements such as poorly kept or rundown housing. Since it is known that children who live in neighborhoods that lack amenities and which have detracting elements may have adverse health outcomes [19,46], and that TSE is independently associated with pediatric physical and mental health morbidity [4,47], these results indicate the need for interventions in these children. Further, the current study identified that TSE, including THS exposure alone, is a risk factor that should be targeted in future community interventions. Thus, addressing TSE reduction and neighborhood improvements should be a priority for children with TSE living in at-risk neighborhoods as such strategies may decrease children’s future tobacco use trajectory and health outcomes. Thus, it is recommended that pediatric health educators screen children for TSE at every opportunity and assess for other risk factors including housing status as this may target children in need of urgent intervention. Parents of children who are identified as being exposed to tobacco smoke should be provided with counseling on ways to quit tobacco use and decrease their child’s TSE and given information on free state- and national-level services and resources to help them achieve these goals (e.g., state tobacco Quitlines) [48].

## Figures and Tables

**Table 1:** Sociodemographic covariates and child home TSE status among U.S. children 6–11 Years Old, 2018–2019 NSCH.

		Child Home TSE Status			
	Overall (N = 17,300)	No TSE (*n* = 14,724)	THS Exposure Alone (*n* = 2278)	SHS and THS Exposure (*n* = 298)	*p*-Value ^b^
Characteristic	*n* (%) ^a^	*n* (%) ^a^	*n* (%) ^a^	*n* (%) ^a^	
**Child Age, M (SE)**	8.56 (0.03)	8.54 (0.03)	8.60 (0.07)	8.64 (0.18)	0.670
**Child Sex**					0.674
Male	8994 (50.9)	7670 (51.1)	1157 (49.2)	167 (51.8)	
Female	8306 (49.1)	7054 (48.9)	1121 (50.8)	131 (48.2)	
**Child Race/Ethnicity**					**<0.001**
Non-Hispanic White	11,854 (50.7)	10,006 (49.4)	1639 (57.3)	209 (63.5)	
Non-Hispanic Black	1105 (12.9)	952 (13.2)	108 (10.0)	45 (21.8)	
Hispanic	2124 (25.2)	1864 (26.0)	248 (22.4)	12 (6.0)	
Non-Hispanic Other or Multiracial	2217 (11.2)	1902 (11.4)	283 (10.3)	32 (8.7)	
**Parent Education Level**					**<0.001**
≤High school graduate/equivalent	2651 (27.7)	1927 (25.4)	586 (39.0)	138 (56.9)	
Some College	4204 (22.4)	3192 (20.5)	887 (32.6)	125 (34.2)	
≥College Degree	10,445 (49.9)	9605 (54.1)	805 (28.4)	35 (8.9)	
**Family Household Structure**					**<0.001**
Two parents, currently married	11,884 (64.2)	10,582 (66.7)	1207 (53.2)	95 (24.6)	
Two parents, not currently married	1231 (8.4)	884 (7.9)	298 (11.4)	49 (12.0)	
Single parent	3310 (21.2)	2,617 (19.9)	586 (27.7)	107 (36.3)	
Other family type	875 (6.2)	641 (5.5)	187 (7.7)	47 (27.1)	
**Family Federal Poverty Level**					**<0.001**
0–199%	5004 (40.5)	3808 (37.5)	985 (54.8)	211 (83.6)	
200–299%	2884 (15.9)	2374 (15.7)	458 (17.7)	52 (10.9)	
300–399%	2498 (12.1)	2204 (12.6)	278 (9.5)	16 (2.7)	
≥400%	6914 (31.5)	6338 (34.2)	557 (18.0)	19 (2.8)	

Abbreviations: NSCH—National Survey on Children’s Health; TSE—tobacco smoke exposure; THS—thirdhand smoke exposure; SHS—secondhand smoke exposure; M—mean; SE—standard error. ^a^ *n* refers to raw counts and percentages are weighted column percent unless noted otherwise. ^b^ Bold font indicates statistical significance *p* < 0.05.

**Table 2:** Child home TSE status and total number of neighborhood amenities among U.S. children 6–11 years old, 2018–2019 NSCH.

	Number of Neighborhood Amenities (Range 0–4)		
	M (SE) ^a^	β	95% CI ^b^
**Child Home TSE Status**			
No TSE	2.82 (0.03)	Ref	Ref
THS Exposure Alone	2.56 (0.06)	**−0.27**	−0.38, −0.15 ***
SHS and THS Exposure	2.42 (0.14)	**−0.41**	−0.69, −0.13 **
**Child Age**	-	0.01	−0.01, 0.03
**Child Sex**			
Male	2.60 (0.06)	Ref	Ref
Female	2.60 (0.06)	−0.007	−0.08, 0.07
**Child Race/Ethnicity**			
Non-Hispanic White	2.27 (0.06)	Ref	Ref
Non-Hispanic Black	2.77 (0.08)	**0.50**	0.38, 0.62 ***
Hispanic	2.68 (0.07)	**0.41**	0.30, 0.52 ***
Non-Hispanic Other or Multiracial	2.68 (0.07)	**0.41**	0.31, 0.51 ***
**Parent Education Level**			
≤High school graduate/equivalent	2.42 (0.07)	**−0.40**	−0.52, −0.27 ***
Some College	2.56 (0.06)	**−0.26**	−0.35, −0.76 ***
≥College Degree	2.82 (0.06)	Ref	Ref
**Family Household Structure**			
Two parents, currently married	2.51 (0.06)	Ref	Ref
Two parents, not currently married	2.71 (0.08)	0.027	−0.16, 0.21
Single parent	2.60 (0.06)	0.13	−0.08, 0.34
Other family type	2.57 (0.10)	−0.06	−0.24, 0.12
**Family Federal Poverty Level**			
0–199%	2.51 (0.06)	**−0.27**	−0.37, −0.16 ***
200–299%	2.53 (0.07)	**−0.24**	−0.35, −0.14 ***
300–399%	2.58 (0.08)	**−0.20**	−0.31, −0.09 ***
≥400%	2.78 (0.07)	Ref	Ref

Abbreviations: NSCH—National Survey on Children’s Health; TSE—tobacco smoke exposure; THS—thirdhand smoke exposure; SHS—secondhand smoke exposure; M—mean; SE—standard error; CI—confidence interval; Ref—reference category. ^a^ M (SE) refers to mean (SE) number of neighborhood amenities (range 0–4). ^b^ Multiple linear regression model adjusting for the covariates of child age, child sex, child race/ethnicity, parent education level, family household structure, and federal poverty level). Bold font indicates statistical significance *p* < 0.05. *** *p* < 0.001. ** *p* < 0.01.

**Table 3:** Child home TSE status and type of neighborhood amenities among U.S. children 6–11 years old, 2018–2019 NSCH.

	Neighborhood Has Sidewalks or Walking Paths	Multivariable Logistic Regression		Neighborhood Has a Park or Playground	Multivariable Logistic Regression		Neighborhood Has Recreation Center, Community Center, or Boys’/Girls’ Club	Multivariable Logistic Regression		Neighborhood Has Library or Bookmobile	Multivariable Logistic Regression	
	*n* (%) ^a^	AOR	95% CI ^b^	*n* (%) ^a^	AOR	95% CI ^b^	*n* (%) ^a^	AOR	95% CI ^b^	*n* (%)	AOR	95% CI ^b^
**Child Home TSE Status**												
No TSE	10,717 (76.3)	Ref	Ref	11,069 (77.1)	Ref	Ref	7098 (51.0)	Ref	Ref	10,050 (69.8)	Ref	Ref
THS Exposure Alone	1450 (65.4)	**0.71**	0.60, 0.85 ***	1530 (66.3)	**0.71**	0.57, 0.87 **	911 (40.0)	**0.75**	0.62, 0.92 **	1370 (60.8)	**0.78**	0.63, 0.95 *
SHS and THS Exposure	152 (51.4)	**0.49**	0.33, 0.72 ***	178 (63.2)	0.73	0.48, 1.10	89 (31.9)	**0.57**	0.38, 0.85 **	164 (60.5)	0.86	0.57, 1.30
**Child Age, M (SE)**	8.57 (0.03)	1.03	0.99, 1.07	8.55 (0.03)	1.00	0.95, 1.04	8.58 (0.04)	1.00	0.99, 1.06	8.55 (0.03)	1.00	0.97, 1.04
**Child Sex**												
Male	6368 (74.7)	Ref	Ref	6582 (74.6)	Ref	Ref	4.200 (49.7)	Ref	Ref	5987 (68.1)	Ref	Ref
Female	5951 (74.2)	0.97	0.85, 1.10	6.195 (76.4)	1.10	0.95, 1.27	3898 (48.8)	0.96	0.85, 1.09	5597 (68.6)	1.03	0.90, 1.17
**Child Race/Ethnicity**												
Non-Hispanic White	7992 (67.6)	Ref	Ref	8526 (73.0)	Ref	Ref	5156 (44.1)	Ref	Ref	7824 (66.5)	Ref	Ref
Non-Hispanic Black	875 (80.8)	**2.37**	1.89, 2.97 ***	829 (78.4)	**1.58**	1.25, 1.99 ***	640 (59.8)	**2.15**	1.74, 2.65 ***	780 (71.9)	**1.43**	1.14, 1.80 **
Hispanic	1683 (81.5)	**2.52**	2.03, 3.13 ***	1643 (75.9)	**1.46**	1.15, 1.84 **	1114 (51.3)	**1.60**	1.31, 1.95 ***	1426 (68.4)	**1.31**	1.07, 1.61 **
Non-Hispanic Other or Multiracial	1769 (82.7)	**2.31**	1.89, 2.83 ***	1779 (82.5)	**1.73**	1.41, 2.13 ***	1188 (55.5)	**1.57**	1.32, 1.85 ***	1554 (73.5)	**1.38**	1.14, 1.66 ***
**Parent Education Level**												
≤High school graduate/equivalent	1675 (70.8)	**0.65**	0.53, 0.80 ***	1729 (68.6)	**0.54**	0.43, 0.66 ***	1051 (43.8)	**0.67**	0.55, 0.82 ***	1540 (61.7)	**0.61**	0.50, 0.75 ***
Some College	2798 (71.3)	**0.76**	0.65, 0.90 **	2907 (71.4)	**0.64**	0.54, 0.75 ***	1.749 (45.0)	**0.75**	0.64, 0.88 ***	2645 (65.8)	**0.75**	0.64, 0.88 ***
≥College Degree	7846 (78.0)	Ref	Ref	8141 (81.1)	Ref	Ref	5298 (54.1)	Ref	Ref	7399 (73.4)	Ref	Ref
**Family Household Structure**												
Two parents, currently married	8522 (75.1)	Ref	Ref	8876 (76.0)	Ref	Ref	5624 (48.9)	Ref	Ref	8059 (68.5)	Ref	Ref
Two parents, not currently married	889 (74.8)	1.06	0.81, 1.40	941 (78.1)	**1.44**	1.08, 1.94 *	565 (51.6)	1.30	1.0, 1.69	809 (69.6)	1.28	0.97, 1.69
Single parent	2355 (74.9)	1.04	0.88, 1.24	2377 (74.0)	1.11	0.91, 1.36	1521 (49.1)	1.11	0.93, 1.32	2156 (68.5)	1.18	0.98, 1.42
Other family type	543 (66.7)	0.79	0.60, 1.05	583 (71.7)	1.09	0.81, 1.46	388 (49.2)	1.30	0.97, 1.75	560 (66.1)	1.15	0.83, 1.58
**Family Federal Poverty Level**												
0–199%	3344 (72.8)	**0.73**	0.60, 0.88 **	3457 (71.5)	**0.71**	0.59, 0.87 ***	2103 (45.8)	**0.68**	0.57, 0.81 ***	3135 (64.8)	**0.74**	0.62, 0.88 ***
200–299%	2000 (71.9)	**0.73**	0.61, 0.89 **	2038 (73.7)	**0.76**	0.62, 0.94 **	1231 (45.7)	**0.71**	0.59, 0.85 ***	1832 (65.6)	**0.75**	0.62, 0.90 **
300–399%	1750 (72.0)	**0.72**	0.60, 0.86 ***	1804 (75.3)	**0.77**	0.63, 0.94 *	1108 (47.9)	**0.76**	0.63, 0.92 **	1639 (69.7)	0.86	0.70, 1.05
≥400%	5225 (78.9)	Ref	Ref	5478 (81.6)	Ref	Ref	3656 (55.9)	Ref	Ref	4978 (74.1)	Ref	Ref

Abbreviations: Abbreviations: NSCH—National Survey on Children’s Health; TSE—tobacco smoke exposure; THS—thirdhand smoke exposure; SHS—secondhand smoke exposure; M—mean; SE—standard error; AOR—adjusted odds ratio; CI—confidence interval; Ref—reference category. ^a^ *n* refers to unweighted sample size and % refers to weighted row percent, unless otherwise noted. ^b^ Four separate multivariable logistic regression models with the reference category as “no” and adjusting for the covariates. Bold font indicates statistical significance *p* < 0.05. *** *p* < 0.001. ** *p* < 0.01. * *p* < 0.05.

**Table 4:** Child home TSE status and total number of detracting neighborhood elements among U.S. children 6–11 years old, 2018–2019 NSCH.

	Total Number of Detracting Elements in Child’s Neighborhood (Range 0–3)		
	M (SE) ^a^	β	95% CI ^b^
**Home TSE Status**			
No TSE	0.43 (0.02)	Ref	Ref
THS Exposure Alone	0.51 (0.04)	0.08	−0.01, 0.16
SHS and THS Exposure	0.67 (0.10)	**0.24**	0.05, 0.43 *
**Child Age, M (SE)**	-	**−0.02**	−0.39, −0.01 ***
**Child Sex**			
Male	0.54 (0.04)	Ref	Ref
Female	0.53 (0.04)	0.01	0.04, 0.06
**Child Race/Ethnicity**			
Non-Hispanic White	0.44 (0.04)	Ref	Ref
Non-Hispanic Black	0.64 (0.05)	**0.20**	0.12, 0.29 ***
Hispanic	0.53 (0.05)	**0.09**	0.01, 0.18 *
Non-Hispanic Other or Multiracial	0.54 (0.05)	**0.10**	0.03, 0.18 **
**Parent Education Level**			
≤High school graduate/Equivalent	0.61 (0.05)	**0.15**	0.07, 0.22 ***
Some College	0.53 (0.04)	**0.07**	0.01, 0.12 *
≥College Degree	0.47 (0.04)	Ref	Ref
**Family Household Structure**			
Two parents, currently married	0.53 (0.04)	Ref	Ref
Two parents, not currently married	0.56 (0.06)	**0.14**	0.03, 0.25 *
Single parent	0.60 (0.05)	0.11	−0.02, 0.23
Other family type	0.46 (0.05)	0.07	−0.24, 0.17
**Family Federal Poverty Level**			
0–199%	0.64 (0.04)	**0.18**	0.11, 0.25 ***
200–299%	0.57 (0.05)	**0.12**	0.05, 0.19 **
300–399%	0.48 (0.05)	0.03	−0.04, 0.10
≥400%	0.45 (0.05)	0	Ref

Abbreviations: NSCH—National Survey on Children’s Health; TSE—tobacco smoke exposure; THS—thirdhand smoke exposure; SHS—secondhand smoke exposure; M—mean; SE—standard error; CI—confidence interval; Ref—reference category. ^a^ M (SE) refers to mean (SE) number of detracting elements (range 0–3). ^b^ Multiple linear regression model adjusting for the covariates of child age, child sex, child race/ethnicity, parent education level, family household structure, and federal poverty level). Bold font indicates statistical significance *p* < 0.05. *** *p* < 0.001. ** *p* < 0.01. * *p* < 0.05.

**Table 5:** Child home TSE status and type of detracting neighborhood elements among U.S. children 6-11 years old, 2018-2019 NSCH.

	Litter or Garbage	Multivariable Logistic Regression		Poorly Kept or Rundown Housing	Multivariable Logistic Regression		Vandalism	Multivariable Logistic Regression	
	*n* (%) ^a^	AOR	95% CI ^b^	*n* (%) ^a^	AOR	95% CI ^b^	*n* (%) ^a^	AOR	95% CI ^b^
**Home TSE Status**									
No TSE	2.328 (19.8)	Ref	Ref	1670 (11.9)	Ref	Ref	787 (6.9)	Ref	Ref
THS Exposure Alone	462 (25.0)	1.26	0.95, 1.57	388 (18.5)	**1.39**	1.06, 1.82 *	184 (8.5)	1.10	0.79, 1.53
SHS and THS Exposure	84 (35.4)	**1.80**	1.10, 2.95 *	84 (25.3)	**1.65**	1.07, 2.54 *	41 (14.0)	1.63	0.92, 2.89
**Child Age, M (SE)**	8.42 (0.06)	**0.93**	0.88, 0.98 **	8.41 (0.07)	**0.94**	0.89, 0.99 *	8.37 (0.10)	**0.92**	0.86, 0.99 *
**Child Sex**									
Male	1491 (21.1)	Ref	Ref	1116 (13.6)	Ref	Ref	510 (7.3)	Ref	Ref
Female	1383 (20.4)	0.98	0.82, 1.16	1026 (12.5)	0.93	0.77, 1.12	503 (7.1)	0.99	0.76, 1.30
**Child Race/Ethnicity**									
Non-Hispanic White	1651 (14.2)	Ref	Ref	1422 (11.9)	Ref	Ref	583 (5.1)	Ref	Ref
Non-Hispanic Black	297 (31.5)	**2.18**	1.72, 2.76 ***	178 (17.6)	1.21	0.93, 1.59	102 (11.9)	**1.83**	1.29, 2.61 ***
Hispanic	495 (27.7)	**1.83**	1.44, 2.33 ***	293 (13.8)	0.88	0.66, 1.19	184 (8.6)	1.30	0.87, 1.93
Non-Hispanic Other or Multiracial	467 (22.9)	**1.79**	1.43, 2.24 ***	249 (11.3)	0.92	0.69, 1.23	144 (7.8)	**1.50**	1.02, 2.22 *
**Parent Education Level**									
≤High school graduate/Equivalent	636 (29.3)	**1.40**	1.09, 1.80 **	486 (18.8)	**1.61**	1.24, 2.08 ***	253 (10.9)	**1.50**	1.06, 2.10 *
Some College	828 (23.4)	**1.25**	1.01, 1.54 *	669 (14.9)	**1.31**	1.06, 1.63 *	281 (7.7)	1.17	0.85, 1.60
≥College Degree	1410 (14.9)	Ref	Ref	987 (9.0)	Ref	Ref	479 (4.9)	Ref	Ref
**Family Household Structure**									
Two parents, currently married	1720 (17.3)	Ref	Ref	1298 (11.3)	Ref	Ref	577 (5.9)	Ref	Ref
Two parents, not currently married	289 (27.1)	1.22	0.89, 1.67	205 (15.5)	1.02	0.73, 1.42	116 (8.4)	1.02	0.69, 1.52
Single parent	719 (28.5)	**1.26**	1.01, 1.58 *	519 (16.6)	1.07	0.83, 1.37	263 (10.8)	1.27	0.92, 1.77
Other family type	146 (21.7)	0.83	0.60, 1.17	120 (15.5)	0.90	0.64, 1.27	57 (7.1)	0.76	0.48, 1.19
**Family Federal Poverty Level**									
0–199%	1214 (28.7)	**1.76**	1.37, 2.26 ***	924 (18.0)	**1.98**	1.48, 2.63 ***	458 (10.4)	**1.83**	1.20, 2.90 **
200–299%	532 (21.1)	**1.51**	1.16, 1.97 **	416 (14.3)	**1.73**	1.27, 2.35 ***	182 (7.6)	1.60	1.0, 2.56
300–399%	342 (15.9)	1.24	0.93, 1.66	260 (9.5)	1.21	0.87, 1.69	111 (3.9)	0.90	0.53, 1.51
≥400%	786 (12.3)	Ref	Ref	542 (7.4)	Ref	Ref	262 (4.1)	Ref	Ref

Abbreviations: NSCH—National Survey on Children’s Health; TSE—tobacco smoke exposure; THS—thirdhand smoke exposure; SHS—secondhand smoke exposure; M—mean; SE—standard error; CI—confidence interval; Ref—reference category. ^a^ *n* refers to unweighted sample size and % refers to weighted row percent, unless otherwise noted. ^b^ Three separate multivariable logistic regression models with the reference category as “no” and adjusting for the covariates. Bold font indicates statistical significance *p* < 0.05. *** *p* < 0.001. ** *p* < 0.01. * *p* < 0.05.

## Data Availability

Data are publicly available on the NSCH website at https://www.childhealthdata.org/learn-about-the-nsch/NSCH (accessed on 24 January 2022).
